# The role of health promotion practitioners during disease outbreaks in the OR Tambo District, South Africa

**DOI:** 10.4102/jphia.v17i1.1650

**Published:** 2026-08-27

**Authors:** Mzwanele Toni, Nongiwe L. Mhlanga, Sibusiso C. Nomatshila

**Affiliations:** 1 School of Public Health, Faculty of Medicine and Health Sciences, Walter Sisulu University, Mthatha, South Africa

**Keywords:** health promotion, outbreaks, health promotion practitioner, role, COVID-19, listeriosis, health education, prevention

## Abstract

**Background:**

In South Africa, infectious disease outbreaks are common and pose severe public health threats. To mitigate outbreaks, health promotion plays a significant role. However, the role of health promotion practitioners remains obscure.

**Aim:**

To describe the role of health promotion practitioners during outbreaks in the OR Tambo District.

**Setting:**

The study was conducted in the Oliver Reginald (OR) Tambo District, a predominantly rural area in the Eastern Cape province of South Africa.

**Methods:**

The study used a qualitative descriptive-phenomenological design. The population comprised health promotion practitioners with a tertiary qualification. These were selected using snowball sampling until data saturation. Data were collected using individual face-to-face and telephonic interviews. Thematic analysis was used to analyse data.

**Results:**

The sample consisted of 14 participants, with the majority (57.1%, *n* = 8) being female. Three themes were identified. Firstly, the implementation role in planning and health education. This was supported by three subthemes: (1) planning and organising, (2) health education collaboration with government departments and (3) multidisciplinary collaborative health education. Secondly, the theme was research, monitoring and evaluation, supported by two subthemes: (1) surveillance and assessment and (2) monitoring and evaluation. Thirdly, the theme, advocacy, was supported by the subtheme advocacy for health services.

**Conclusion:**

The roles of health promotion practitioners during outbreaks were aligned with the national strategy and could be optimised by inclusion in training and planning activities.

**Contribution:**

The study contributes to the efficient management of infectious disease outbreaks by clarifying the role of health promotion practitioners.

## Introduction

Globally, Africa reported the highest number of infectious disease outbreaks in 2018, with 96 new outbreaks.^1^ This high number of infectious disease outbreaks is partly because of climate change, which contributes to the climatic suitability of vector-borne diseases^2^ and increased travel.^3,4^ In South Africa, major infectious disease outbreaks, including the listeriosis outbreak from 2017 to 2018,^5^ and coronavirus disease 2019 (COVID-19)^6^ have been reported, posing severe public health threats. During outbreaks, such as the COVID-19 pandemic, community health workers are often assigned community-level duties to prevent and manage them.^7^ In addition, other health workers, such as nurses and environmental health officers, perform health promotion activities and collaborate with health promotion practitioners – professionals who focus on the practical socio-environmental determinants of health.^8,9^ Health promotion activities in South Africa are delivered at the primary healthcare level^9,10^ and broadly encompass training, providing health education, research, policy coordination and community development.^9^ Given that different health workers perform health promotion activities in contexts where infectious disease outbreaks frequently occur, it is necessary to clarify the roles of health promotion practitioners to reduce role ambiguity and foster efficient team effort. Therefore, the purpose of this study was to describe the role of health promotion practitioners in infectious disease outbreaks in the OR Tambo District.

The Ottawa Charter of 1986^11^ facilitated the inclusion of the health promotion agenda in health policies across several countries.^12^ Since then, health promotion has grown as a discipline in some countries,^8,12^ with advocacy^13,14,15^ for active inclusion of health promotion during major disease outbreaks, such as the COVID-19 pandemic, becoming increasingly prominent. During outbreaks, health promotion practitioners can assume tasks such as improving preventive behaviour through health education.^15^ This need for health promotion during outbreaks is essential to inform individual behaviour change, develop interventions to promote health and inform health promotion policies during outbreaks.^13^

Several studies^16,17,18^ have examined the impact of health promotion in infectious disease control, noting how health promotion practitioners work with communities through mobilisation, health education programmes and empowerment initiatives.^16^ For example, in Iraq, health promotion was acknowledged as instrumental in strengthening COVID-19 vaccination through engaging the community by delivering health education to encourage vaccine uptake.^17^ Although health promotion is acknowledged as instrumental in the Iraq study, notably, the teams responsible for the success of the vaccination campaign comprised vaccinators, community mobilisers, among other team members, such as data entry clerks and registrars.^17^ Similarly, in Singapore and Malaysia, migrant communities who lived in overcrowded dormitory facilities benefited from health promotion activities during the COVID-19 pandemic. In this regard, health promoters encouraged the implementation of hygiene measures in the dormitories to reduce the spread of COVID-19, while the government quarantined the dormitories, thus also illustrating how government policy can support health promotion initiatives.^18^ These studies from Iraq, Singapore and Malaysia, although highlight the importance of health promotion during outbreaks, also underscore the multidisciplinary nature of health promotion and raise the question: *Do we need trained and professional dedicated health promotion practitioners during outbreaks?*

In South Africa,^19^ like in other low-income to middle-income countries,^12^ the role of health promotion practitioners remains poorly defined and often dominated by health professions with curative roles.^19^ Despite this, there are South African studies^10,19^ that acknowledge the work implemented by dedicated health promotion practitioners. Boboko et al.,^10^ in their study in the Dr. Kenneth Kaunda District in the North West province of South Africa, note that the work of health promotion practitioners in South Africa is often marred by insufficient resources, inadequate managerial monitoring and supervision and inadequate health promotion structures.^10^ The poorly defined role of health promotion practitioners in South Africa also occurs in a context marked by several infectious disease outbreaks. For example, between 2013 and 2017, 327 foodborne disease outbreaks were reported in South Africa, resulting in 8680 hospital visits.^20^ Given the high burden of infectious disease outbreaks, the importance of health promotion in outbreak mitigation and the poorly defined role of professional health promotion practitioners in South Africa, this study sought to describe the role of health promotion practitioners in disease outbreaks in the OR Tambo District in South Africa. This description will clarify the roles of health promotion practitioners, facilitating effective health promotion during infectious disease outbreaks.

To describe the role of health promotion practitioners, the study was framed in accordance with the National Health Promotion Policy and Strategy 2015–2019, which was the national framework in use at the time.^9^ This framework was selected as it guides the activities of health promoters. The National Health Promotion Policy and Strategy 2015–2019 outlines six areas of action for health promotion at three different levels in South Africa. The levels include national, provincial and district or sub-district levels. The areas of action are categorised into six broad areas: (1) advocacy and communication; (2) policy and regulatory framework; (3) leadership; (4) implementation; (5) research, monitoring and evaluation and (6) skills development and capacity building.^9^

## Research methods and design

The study used a qualitative research approach and a descriptive-phenomenological design. The researchers selected this design to ensure a complete description of the roles that health promotion officers play during outbreaks. The study was conducted in the OR Tambo District located in the Eastern Cape province of South Africa. In 2022, the district had a population of 1 501 702 and was the fourth largest in South Africa.^21^ The OR Tambo District comprises five local municipalities, is predominantly rural, and most (83.27%) people live below the poverty line.^21^

The study population comprised health promotion practitioners working in the district at the time of data collection. The study included only health promotion practitioners who had completed a tertiary qualification in health promotion and were working in the OR Tambo District for at least 1 year and had a role in an outbreak. The study excluded health promotion practitioners who were unwilling to participate, those without a role in prior infectious disease outbreaks and those with fewer than 1 year of work experience. Snowball sampling was employed to select study participants because of the scarcity of information on the population size and the difficulty in locating health promotion practitioners across various community settings. The researcher began by locating health promotion practitioners whom they knew and were closest to the researcher. Thereafter, the identified health promotion practitioners referred other practitioners in the district. The sample size was determined by data saturation.

Data were collected using individual interviews with a semi-structured interview guide by the researcher Mzwanele Toni. At the time of data collection, the researcher, Mzwanele Toni, had also been trained as a health promotion practitioner, worked in quality assurance at a tertiary educational institution and had no personal or professional relationship with the participants. A pilot study was first conducted in April 2021. After the pilot study, the researchers included a screening question about whether the health promotion practitioners had worked during an infectious disease outbreak. Data collection was then conducted in May and June 2021. All interviews were conducted in isiXhosa, as preferred by the participants, and each lasted approximately 40 min. One interview was conducted by telephone, and the remaining interviews were conducted face to face in various community settings where health promotion practitioners worked. These included private rooms in primary healthcare facilities and schools. The interview questions were designed to elicit the tasks performed during outbreaks, the outbreaks in which they worked and the training they received during these outbreaks. Interview questions also included the health promotion practitioners’ experiences with the tasks they carried out during outbreaks, their work experiences during outbreaks, their perceptions of their capacity to manage outbreaks, with whom they collaborated and how and their perceptions of the impact of their tasks during outbreaks.

Data were analysed using Braun and Clark’s six steps of thematic analysis.^22^ The researcher Nongiwe L. Mhlanga analysed data with the assistance of a co-coder, Mzwanele Toni. NVivo version 9 was used to analyse data, using an inductive approach. This entailed transcribing and translating the collected data, then using NVivo to generate the codebook, which was based on four transcripts selected by the researchers. Themes were then searched across the remaining dataset and reviewed. The generated themes were named, and a report was produced. While collecting and analysing data, the researchers reflected on their roles as researchers. To ensure trustworthiness, Lincoln and Guba’s framework^23^ was used, with member checking conducted to ensure credibility and dependability. This was achieved by presenting transcripts to seven participants, who confirmed that the findings accurately reflected the discussions during the interviews. After this, no further changes were made. Direct quotes from participants were also included in the findings to ensure credibility. Credibility was further ensured by analysing data with a co-coder (Mzwanele Toni), who also assisted in developing a codebook. Dependability and confirmability were also guaranteed by detailing how the study was conducted. The researchers also described the study setting in detail to guarantee transferability and confirmability.

### Ethical considerations

Ethical clearance to conduct this study was obtained from the Walter Sisulu University, Faculty of Health Sciences, Postgraduate Education, Training, Research, and Ethics Unit, with approval number – 024/2021. The researchers adhered to the Declaration of Helsinki ethical standards for conducting research with human participants.^29^ Written consent was obtained from all participants individually, and confidentiality was maintained by using pseudonyms and conducting interviews in a private setting. The participants were all informed of measures to protect the data collected.

## Results

The sample consisted of 14 health promotion practitioners working in the OR Tambo District. Most (57.1%, *n* = 8) participants were female, while males comprised 42.9% of the sample. Participants’ ages ranged from 26 years to 54 years, with an average age of 36.1 years (standard deviation [s.d.] ± 7.8). The majority (64.3%, *n* = 9) of participants worked in rural settlements, while 35.7% worked in urban settlements. Participants’ work experience ranged from 1 year to 15 years. Table 1, which follows, shows the sample demographic characteristics.

**TABLE 1:** Sample demographic characteristics.

Participant	Age (years)	Gender	Type of settlement (rural or urban)	Years of experience	Highest level of education
P1	30	Female	Rural	4 years	Bachelor’s degree
P2	34	Male	Urban	More than 1 year	Bachelor’s degree
P3	34	Male	Not specified	3 years	Bachelor’s degree
P4	41	Male	Rural	6 years	Bachelor’s degree
P5	37	Female	Rural	5 years	Bachelor’s degree
P6	31	Female	Rural	2 years	Bachelor’s degree
P7	26	Male	Rural and urban	More than 1 year	Bachelor’s degree
P8	47	Female	Rural	15 years	Bachelor’s degree
P9	35	Female	Urban	1 year	Bachelor’s degree
P10	29	Male	Rural	1 year	Bachelor’s degree
P11	45	Male	Urban	3 years	Postgraduate degree
P12	54	Female	Rural	9 years	Bachelor’s degree
P13	35	Female	Rural	3 years	Bachelor’s degree
P14	27	Female	Semi-rural	4 years	Postgraduate degree

### Themes

Three themes emerged from the study’s findings: (1) implementation role: Planning and collaborative health education; (2) research, monitoring and evaluation role during outbreaks and (3) advocacy and communication.

### Theme 1: Implementation role: Planning and collaborative health education

Participants outlined their roles in outbreak planning and their collaboration in providing health education. This collaboration involved providing health education to health workers within multidisciplinary teams, as well as collaboration with government departments and non-governmental organisations (NGOs). Three subthemes supported this subtheme.

#### Subtheme 1.1: Planning during outbreaks

The participants observed how they planned and organised activities to contain the outbreak. The planning and organising tasks, as noted by P1, P3 and P4, required a shift in focus from day-to-day duties to urgent priorities. Participants described how they plan activities; however, they were unable to contribute to the broader health promotion plan in the Department of Health:

‘My duties involve conducting awareness campaigns, planning, and implementing the programmes for the prevention and control of disease outbreaks … because I need to adjust to the current situation and focus on the interventions to handle that outbreak.’ (P3, 34-year-old, male, 3 years’ experience)

A similar assertion was made by another participant, who highlighted that although they planned for outbreaks, their plans often differed from the broader organisational development plans:

‘The problem is that we don’t have an equal health care plan or disaster management plan because if we had it, we would not be facing some of the health problems we face now.’ (P4, 41-year-old, male, 6 years’ experience)

Although P3 describes how they plan to mitigate the disease outbreaks, they also noted that their plans are often not included in the broader health promotion plan of the Department of Health, as a bottom-up approach was not used to develop the department’s plan. This was noted from the quote:

‘They [*management*] should use a bottom-up approach because it will allow everyone to be part of planning and implementation of health programmes.’ (P3, 34-year-old, male, 3 years’ experience)

On the contrary, P1 described how they were also involved in planning and organising awareness sessions during outbreaks. In this regard, the participant described how their management was supportive and would visit communities based on the feedback provided by the participant:

‘I am responsible for conducting community awareness, community mobilisation, planning health events, and community development projects. When there is an outbreak, my work responsibilities change because we’ve had to drop everything and attend to that outbreak. We organised the listeriosis campaign, where we were educating them about listeriosis, its causes, prevention, and management. They [*management*] take every outbreak seriously; they do community visits to ensure that the community is safe and do take our feedback into consideration.’ (P1, 30-year-old, female, 4 years’ experience)

Participants highlighted how they planned and organised community awareness and mobilisation urgently, during outbreaks. This was supported by management structures that took feedback from health promotion practitioners seriously, although in the cases of P3 and P4, their planning was sometimes not included in the broader plans.

#### Subtheme 1.2: Multidisciplinary collaboration to provide health education

Health promotion activities, such as providing health education, are multidisciplinary, and participants P1, P3, P4 and P14 articulated how they collaborated with other health professionals in providing health education during outbreaks. In this description, participants noted key messages they articulated to community members, focusing on prevention, and indicated that they would collaborate with health workers, such as environmental health practitioners and nurses:

‘We were working together with environmental health practitioners … even during COVID-19, we went to the communities to educate them about the causes, how they can prevent themselves from being infected, and how to handle people who are already infected.’ (P1, 30-year-old, female, 4 years’ experience)

The researcher also asked P1 whether they perceived that community members abided by the health education provided. In response, the participant opined:

‘The community members usually listen and follow everything they are told in order to bring their community back to health. We do not go back to the same community for the same issue.’ (P1, 30-year-old, female, 4 years’ experience)

Similarly, P3 reiterated the nature of health education by stating:

‘We educate people about the outbreak, ensuring that they understand the main cause, the prevention methods, and how to control that certain outbreak.’ (P3, 34-year-old, male, 3 years’ experience)

Participant P4 also indicated that he had been working with nurses and other health professionals and recalled an information campaign they had conducted:

‘We only had one information campaign in Alice during COVID-19, where we felt at least involved with others.’ (P4, 41-year-old male, 6 years’ experience)

In addition, P4 described other channels they had used to provide health education:

‘[*W*]e render information to the community through all the models of communication, to mention a few, radio, television, clinic posters, and billboards.’ (P4, 41-year-old male, 6 years’ experience)

Although participants acknowledged working in a multidisciplinary team, some, such as P14 and P4, shared that the collaborative role among health workers during outbreaks could be improved. Participant 14 had broadly stated that they worked with other health workers:

‘Everyone is taking the current outbreak [*COVID-19*] seriously, so they respond positively. We are working together with other health professionals to beat this pandemic.’ (P14, 27-year-old, female, 4 years’ experience)

When asked how their skills could be improved to better manage infectious disease outbreaks, P14 observed that their collaborative efforts during pandemics could be improved through training. In this regard, the participant stated:

‘… [*A*]nd lastly, we need to be trained on how we can collaboratively work with other health professionals.’ (P14, 27-year-old, female, 4 years’ experience)

Similarly, P4 perceived that their collaborative role was not reciprocated, as they were excluded from training with other health workers who also carried out health promotion activities:

‘Depending on the outbreak present at that moment, the department should allow us to attend all the training that is offered to the nurses, such as conducting screenings, presentations.’ (P4, 41-year-old male, 6 years’ experience)

In summary, health promotion practitioners work collaboratively with health workers across various health disciplines during outbreaks. This collaborative role is evident in health education tasks, where they focus on informing community members about prevention. The health promotion practitioners were limited in their collaborative role by a lack of training on collaboration during outbreaks, as well as exclusion, despite other disciplines receiving training during these periods.

#### Subtheme 1.3: Collaboration with government departments and non-governmental organisations to provide health education

The collaboration to provide health education could also be extended to other government departments, such as the South African Social Security Agency (SASSA), the Department of Education, the Department of Social Services, and Home Affairs (police). In addition, the participants collaborated with NGOs and local political structures to deliver health education. Participants P6, P8, P9 and P13 described the collaboration with other departments:

‘The campaigns that we do in Mhlonhlo are in collaboration with other departments. When they are hosting, we get invitations and address the certain parts that require health promotion.’ (P9, 35-year-old, female, 1 year experience)

The participant P9 further stated the different departments they worked with:

‘… [*W*]e do work with various departments such as the educational department and social development.’ (P9, 35-year-old, female, 1 year experience)

Other participants, P13 and P8, reiterated the issue of collaboration with government departments and municipalities:

‘I do collaborate with other departments, such SASSA department and the Department of Home Affairs.’ (P13, 35-year-old, female, 3 years’ experience)

‘… [*B*]ut we work together for the better health of an individual, such as social development, education, and municipalities.’ (P8, 47-year-old, female, 15 years’ experience)

Participants P1, P4, P6, P10 and P11 explained that one of the tasks they performed collaboratively with other departments was providing health education. In this regard, P6 opined:

‘I am only working for the Department of Health, but I work in collaboration with social development when they are hosting awareness campaigns.’ (P6, 31-year-old, female, 2 years’ experience)

Participant P11 also observed that they collaborated with local political leadership during the COVID-19 pandemic to provide health education:

‘We faced COVID-19, and it was very serious, especially in the Qunu community a lot of people died. My role was to make the community aware of the disease that may affect them through campaigns, working with community members, ward councillors to bring about better health.’ (P11, 45-year-old, male, 3 years’ experience)

In addition, when further probed on the nature of messaging they provided during awareness campaigns that required collaboration with other departments and health workers, P10 gave an example of prevention strategies and reinforcing regulations on the limited number of people who can attend funeral gatherings during the COVID-19 pandemic, while P3 described how they educated people on the causes, prevention methods and control measures:

‘We are currently working with the Department of Education, police and NGO’s … most people do not take COVID-19 seriously if the community leaders were taking seriously this disease people were going to follow their steps for example there is a certain number of people allowed to attend the funerals, but they don’t follow any of the regulations, My duty is to the communities to deliver health education on this … my duty to communities is to deliver health education on certain disease prevention strategies, how to address health issues.’ (P10, 29-year-old, male, 1 year experience)

Based on the participants’ shared experiences above, health promotion practitioners collaborate with various government departments, political structures and NGOs to provide health education during outbreaks. The messages reinforce government regulations and provide information on prevention.

### Theme 2: Research and monitoring role during outbreaks

The participants described the research and monitoring roles they implemented during outbreaks. This theme was supported by two subthemes: Surveillance and assessment and outbreak monitoring.

#### Subtheme 2.1: Surveillance and assessments during outbreaks

The participants P6, P8, P9 and P14, also described how they collaborated with team members to investigate disease outbreaks, which informed outbreak mitigation or provided information for health education.

In the case of P8, the participant reported that they had also worked collaboratively as part of an outbreak response team. They would investigate the cause of the outbreak to enable timely notification. The quote from P8 and P9 is shown below:

‘I research that disease so that we can go to people with updated information and educate them on how to prevent the disease.’ (P9, 35-year-old, female, 5 years’ experience)

‘In each team, we have an outbreak response team. If there is an outbreak, we go out first to investigate the disease, trying to find the initial cause, and then we take information straight to our authorities so that they can report to the department [*Department of Health*] and relevant stakeholders.’ (P8, 47-year-old, female, 15 years’ experience)

The participant was asked to illustrate a case where they had investigated an outbreak, and they highlighted:

‘[*W*]e experienced so many outbreaks such as diarrhoea in some communities and other community ate the beef that was raw from a vaccinated cow even the current one COVID-19.’ (P8, 47-year-old, female, 15 years’ experience)

This issue of investigating food poisoning from eating raw cow meat was also described by P12, who also said:

‘The disease that we investigated occurred when people ate cow meat that was just vaccinated and it was during the COVID-19 pandemic around March, some people assumed that is caused by dirty water, it was very serious because we were busy with COVID-19 cases and we were forced to split and attend to that outbreak.’ (P12, 54 year-old, female, 9 years’ experience)

The issue of investigating a disease outbreak to capacitate other health workers or departments for the benefit of the community was also described by P14, who stated:

‘… [*Y*]ou must visit the place where there is an outbreak to check what can be the cause of the outbreak to come up with strategies to fight against the outbreak.’ (P14, 27-year-old, female, 4 years’ experience)

In addition, P14 acknowledged that they were working with other health professionals. To investigate the outbreaks, P14 further elaborated how they would have to abandon other research and conduct research aligned with the outbreak to enable information provision for the Department of Health:

‘… [*F*]or example, we were busy with other studies, then there was a COVID-19 pandemic, we had to shift focus and try to find more about COVID-19 to assist the National Department of Health.’ (P14, 27-year-old, female, 4 years’ experience)

#### Subtheme 2.2: Monitoring and evaluation during outbreaks

Participants also explained how they collected data during outbreaks for monitoring purposes. From the participants’ P1, P6 and P12 shared experiences, monitoring entailed assessing the impact of their interventions by recording the number of new cases of the outbreaks. For example, P6 noted how, as part of the outreach team, they presented mortality and morbidity statistics and also trained community health workers to ensure communities provided door-to-door services during outbreaks:

‘My job during outbreaks is to present mortality and morbidity on diseases and to educate, and I am based on outreach. I am doing health education on disease outbreaks to the communities, and I train community health workers since they are the ones who are doing door-to-door visits.’ (P6, 31-year-old, female, 2 years’ experience)

Other participants, P1 and P12, explained how they monitored the number of new cases from outbreaks to assess the impact of their work. The response from P12, who observed that they had to split as a health promotion team because of two outbreaks, described the impact of their work:

‘For example, they [*health promotion practitioners*] are the ones that are managing the progression of COVID-19; their work is noticeable because the cases are decreasing daily.’ (P12, 54-year-old, female, 9 years’ experience)

Likewise, P1 noted a similar issue about the listeriosis outbreak:

‘When we started to visit the communities, especially in Chris Hanni cases were very high. As we were going to address the issue, we managed to end up with zero cases.’ (P1, 30-year-old, female, 4 years’ experience)

Therefore, in summary, during outbreaks, health promotion practitioners monitored the number of people infected.

### Theme 3: Advocacy

Participants also described their roles in advocacy and communication. Noting how they advocated for the provision of health services and empowered communities to ensure the mitigation of outbreaks.

#### Subtheme 3.1: Advocacy for health services during outbreaks

One of the tasks performed by the participants in this study was advocacy, described by participants P5, P13 and P11. This role was fulfilled through health promotion practitioners addressing infectious disease outbreaks by enabling the provision of health services in affected communities. In the case of P13, this role depended on working with primary healthcare facilities and ward councillors to provide curative health services to affected communities. The quote from P13 illustrates how participants advocated for health services:

‘We do campaigns to address any health issues that might harm the health of an individual, and we also advocate for people to bring needed health services’. We make the community aware of every disease that may affect them through campaigns, working with community members, ward councillors to bring about better health.’ (P13, 35-year-old, female, 3 years’ experience)

‘My role is to capacitate the community members about the outbreak. We do awareness campaigns, we address people to reduce stigma among the community members, and we work with the survivors, providing social support to the families that were affected by the disease outbreak. Currently, we are also responsible for COVID-19 vaccine registration.’ (P13, 35-year-old, female, 3 years’ experience)

Likewise, P5 worked in a school environment, where the role included training on human immunodeficiency virus (HIV) and acquired immunodeficiency syndrome (AIDS). However, during the COVID-19 pandemic, this role expanded to support high school learners’ access to primary healthcare services by providing health education, which empowered them:

‘I am working as the Care and Supporter Champion … I am placed in the school am dealing with students from age 16 above, we are teaching them about HIV/AIDS risks, we have had so many COVID-19 cases. The school is the rural areas, and is not given any attention, and the community members do not follow the protective and preventative COVID-19 guidelines. When you think about how we could get COVID-19, we had to work with clinics … my role as the health promoter was to empower the people about the outbreak.’ (P5, 37-year-old, female, 5 years’ experience)

Therefore, from the participants’ responses, health promotion practitioners also advocated for health services through community structures, such as schools and political leadership, to empower communities through health service provision, including primary care services they depended on.

## Discussion

The study found that during infectious disease outbreaks in the OR Tambo District, health promotion practitioners’ tasks include health education, planning and organising, advocacy and communication, monitoring and evaluation and conducting surveillance and assessments. These tasks were mostly performed within teams with other health workers.

The study included 14 participants, all of whom had a minimum qualification of a bachelor’s degree; two participants held a postgraduate qualification. The qualification levels among participants reflect the professional nature of health promotion as a discipline, as evidenced by the high proportion of participants holding university qualifications.^8^ However, the inclusion of participants with only a university degree or higher may limit the applicability of these findings to health promotion practitioners with similar qualifications. This contrasts with other studies in South Africa, which did not include health promotion practitioners with tertiary qualifications.^10,19^ In addition, the study comprised participants who worked primarily in rural areas, reflecting the predominantly rural nature of the OR Tambo District in the Eastern Cape.^21^

Notably, the study found that health promotion practitioners conducted activities during outbreaks that aligned with the activities outlined in the National Health Promotion Policy and Strategy: 2015–2019 at the district level.^9^ These health promotion activities, indicated in the strategy, aligned with three of the six categories of roles and responsibilities.^9^ The activities carried out by the health promotion practitioners are also stated in the Health Promotion Strategy for the African Region, which prioritises the use of a multisectoral approach to ensuring community awareness of preventable diseases.^24^ The study observed how health promotion practitioners in the OR Tambo District worked collaboratively to provide health education with other health disciplines, such as nursing, community health workers and environmental health practitioners. This collaboration with other health workers was not unexpected and affirms the general multidisciplinary nature of health promotion activities.^8,9^ Although their work was collaborative, health education is an inherent function in the discipline of health promotion.^14^ Reviews by Woodall et al.^25^ and Laverack^26^ acknowledge the critical role of health promotion practitioners in providing a reliable source of information during outbreaks, particularly during the COVID-19 pandemic, which was characterised by extensive misinformation.^25^ In this regard, participants such as P1 explained how they planned awareness campaigns and provided reliable information during outbreaks. ‘I am responsible for conducting community awareness’. This collaborative role in providing health education during outbreaks also aligns with a Tanzanian study, which found that during Cholera outbreaks in a rural setting, health promoters are key to delivering health education.^27^ In addition, the prominence of the collaborative role in providing health education may be attributed to the focus of the study only at the district level, where the National Health Promotion Policy and Strategy: 2015–2019^9^ stipulates the formation of epidemic response teams and a multisectoral approach to health promotion, which is reflected in this study. Future studies may also explore the additional role of health promotion practitioners at all levels of service provision, including national and provincial, during outbreaks, to achieve a comprehensive and conclusive understanding of their collaborative role in health education.

Although health promoters provided health education during outbreaks, it was also observed that this collaborative role is constrained by limited inclusion in training, as other health workers with whom they collaborated were trained. Notably, the participants had similar exclusion in planning during outbreaks. This exclusion of health promotion practitioners in training and planning during outbreaks may also reflect the obscurity of health promotion practitioners in South Africa in the broader pool of health professions described by Rwafa-Ponela et al.^19^ and Boboko et al.^10^ However, it is essential to consider that during outbreaks, health promotion plays a crucial role and should be incorporated into outbreak planning.

This study also found that the health promotion officers’ research role encompassed surveillance and assessment, as well as monitoring and evaluation. In case studies presented by Corbin et al.,^28^ the authors note that although health education plays a critical role in disease prevention, it is often insufficient, and other interventions, such as research and monitoring, as well as advocacy, are critical. In the case of the Ebola outbreak in Sierra Leone, one of the critical success factors was the continuous analysis of community-level conditions and the adaptation of health education messaging based on data collected.^28^ Additionally, advocacy played a critical role in ensuring community entry for health promotion interventions in Kenya during the COVID-19 pandemic.^28^ Similarly, in this study, participants reported investigating and documenting the causes of outbreaks and using research to inform their health education. Although health promotion practitioners conduct research in South Africa, there remains a paucity of published literature and institutional reports from a health promotion perspective on outbreak mitigation. Therefore, it is recommended that the Health Promotion Directorate foster this research role by establishing a research repository where case reports on outbreaks can be made available to the broader scientific community.

## Conclusion

The study aimed to describe the role of health promotion practitioners during outbreaks in the OR Tambo District, South Africa. The roles of health promotion practitioners included collaborative health education, planning and organising, advocacy, monitoring, evaluation, surveillance and assessment. These functions of health promotion practitioners align with existing policies, such as the National Health Promotion and Policy Strategy in South Africa at the district level and the Health Promotion Strategy for the African Region. As such, policymakers may use these findings to update existing policies in South Africa and across Africa. Based on these study findings, it is recommended that future studies include health promotion practitioners working at provincial and national levels during outbreaks. It is also recommended that, in the development of outbreak response plans, health promotion officers be included in all aspects of service provision, such as planning to optimise their role in outbreak mitigation, as stated in existing national and regional policies on health promotion. In addition, the study recommends establishing a research repository for health promotion, utilising case reports during outbreaks, to highlight the research role.

The study’s main strengths were a sample that provided adequate information to enable thematic saturation. This was supported by rich narratives that provided adequate insight into the activities of health promotion officers during outbreaks. Another strength was the analytic approach used, which was informed by Lincoln and Guba’s framework. This study, however, had some limitations, including the lack of health promotion practitioners in managerial positions and/or management structures where health promotion practitioners worked, as some responses referred to management structures. The study was also limited by the inclusion of health promotion practitioners who had a minimum of a degree in health promotion, which may exclude the application of these findings to health promotion practitioners with lower-level qualifications. However, this could have been increased by utilising telephonic interviewing and snowball sampling, as applied in the study design, which would have enabled the recruitment of participants with various tertiary-level qualifications and those outside the OR Tambo District. Snowball sampling may also have introduced selection bias, as participants with similar networks may share similar perspectives.
